# Optic Neuritis as Isolated Manifestation of Leptomeningeal Carcinomatosis: A Case Report and Systematic Review of Ocular Manifestations of Neoplastic Meningitis

**DOI:** 10.1155/2013/892523

**Published:** 2013-10-07

**Authors:** Silvia Lanfranconi, Paola Basilico, Ilaria Trezzi, Linda Borellini, Giulia Franco, Vittorio Civelli, Francesco Pallotti, Nereo Bresolin, Pierluigi Baron

**Affiliations:** ^1^Department of Neurological Sciences, Fondazione I.R.C.C.S. Ca' Granda Ospedale Maggiore Policlinico, Dino Ferrari Center, 20122 Milan, Italy; ^2^Department of Neuroradiology and Interventional Neuroradiology, Fondazione I.R.C.C.S. Ca' Granda Ospedale Maggiore Policlinico, 20122 Milan, Italy; ^3^Department of Pathological Anatomy, Fondazione Fondazione I.R.C.C.S. Ca' Granda Ospedale Maggiore Policlinico, 20122 Milan, Italy

## Abstract

*Introduction*. Leptomeningeal carcinomatosis occurs in about 5% of cancer patients. Ocular involvement is a common clinical manifestation and often the presenting clinical feature. *Materials and Methods*. We report the case of a 52-year old lady with optic neuritis as isolated manifestation of neoplastic meningitis and a review of ocular involvement in neoplastic meningitis. Ocular symptoms were the presenting clinical feature in 34 patients (83%) out of 41 included in our review, the unique manifestation of meningeal carcinomatosis in 3 patients (7%). Visual loss was the presenting clinical manifestation in 17 patients (50%) and was the most common ocular symptom (70%). Other ocular signs were diplopia, ptosis, papilledema, anisocoria, exophthalmos, orbital pain, scotomas, hemianopsia, and nystagmus. Associated clinical symptoms were headache, altered consciousness, meningism, limb weakness, ataxia, dizziness, seizures, and other cranial nerves involvement. All patients except five underwent CSF examination which was normal in 1 patient, pleocytosis was found in 11 patients, increased protein levels were observed in 16 patients, and decreased glucose levels were found in 8 patients. Cytology was positive in 29 patients (76%). *Conclusion*. Meningeal carcinomatosis should be considered in patients with ocular symptoms even in the absence of other suggestive clinical symptoms.

## 1. Introduction

Leptomeningeal carcinomatosis results from dissemination of malignant cells to leptomeninges and can be observed in about 5% of patients with malignancies, but it is likely to become more frequent with the increase of life expectancy in cancer patients [1]. Neoplastic cells may spread to the subarachnoid space through (1) arterial circulation or, less frequently, through (2) retrograde flow in venous systems or (3) as a direct consequence of preexisting brain metastases or (4) through migration of neoplastic cells from the original tumor along perineural or perivascular spaces [2, 3]. Clinical manifestations can be highly variable and may affect both central (CNS) and peripheral nervous system (PNS). CNS involvement may lead to generalised symptoms such as seizures, confusion, encephalopathy, or intracranial hypertension as well as, less frequently, to focal neurological symptoms, mainly consisting in hemiparesis or aphasia. PNS involvement may present with lumbar and cervical radiculopathies or cranial neuropathies [2]. Ocular symptoms even in the absence of other clinical symptoms may represent the initial manifestation of meningeal carcinomatosis. Thus, meningeal carcinomatosis should be considered in the differential diagnosis in selected patients even if making the diagnosis is often challenging. Diagnostic tools consist mainly of contrast enhanced CT and MRI and lumbar puncture. Treatment options such as radiation and intrathecal chemotherapy are often palliative with an expected median patient survival of 2 to 6 months [2].

## 2. Case Report

A 52-year old lady was referred to our hospital for acute onset, ten days before hospitalization, of left orbital pain and visual loss associated with mild frontal throbbing headache. As symptoms progressed, she underwent ophthalmological evaluation as outpatient six days after symptoms onset, without evidence of significant visual loss and normal fundus oculi examination. Ocular computed tomography performed at that time was unremarkable. On subsequent ophthalmological evaluation five days later a significant visual loss in the left eye was evident with substantially normal fundus oculi examination. She underwent right mastectomy and hormonal therapy (tamoxifen) for infiltrative breast carcinoma in 2007 followed by chemotherapy with AC (cyclophosphamide and doxorubicin) for six months, followed by letrozole. She was on regular followup.

**Figure 1:**
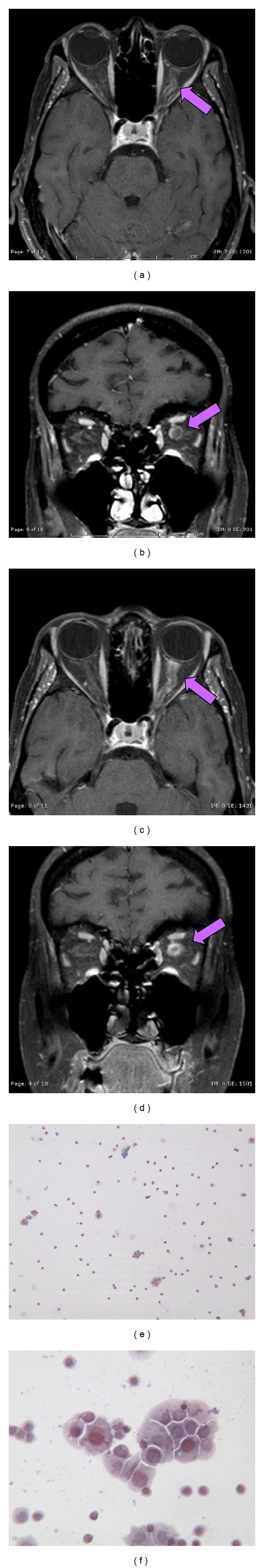
((a)–(d)) Contrast-enhanced cerebral MRI showing diffuse enhancement of the left optic nerve at onset ((a), axial T1; (b), coronal T1) and at 2 months followup ((c), axial T1_SE_FSAT; (d) coronal T1_SE_FSAT). ((e)-(f)) Cerebrospinal fluid cytology showing atypical epithelial cells aggregates.

**Figure 2:**
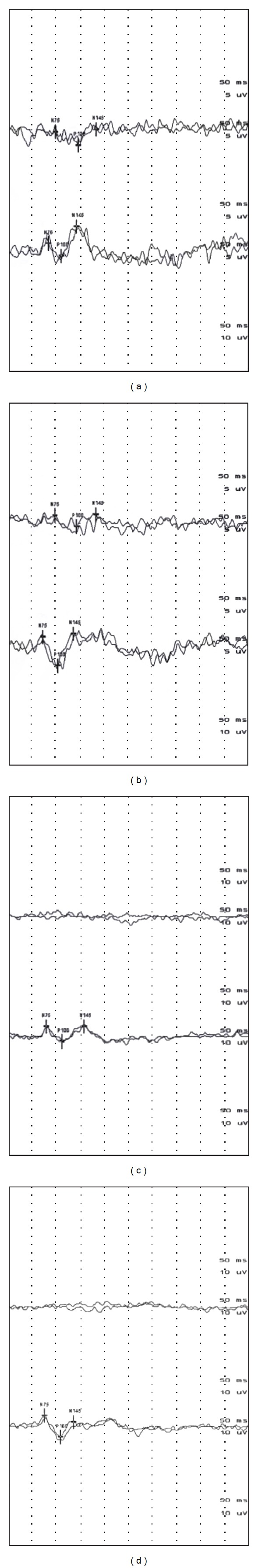
Visual evoked potentials (VEPs) at onset ((a), (b)) and at two months follow-up ((c), (d)). (a)-(b): Response at 15 (a) and 30 (b) sec showing destructured response, reduced amplitude and prolonged latency on the left and normal response on the right side. (c)-(d): Response at 15 (c) and 30 (d) sec revealing absent response on the left and destructured response with normal amplitude and latency on the right side.

On admission in August 2011 neurological examination was normal except for mild anisocoria (left > right) with detectable Markus Gunn sign, left eye visual loss, and global reduction of deep tendon reflexes. She underwent contrast-enhanced cerebral MRI showing nonspecific signal alteration involving frontal subcortical and periventricular white matter and focal contrast enhancement involving the left optic nerve sheath (see Figure 1). VEP revealed destructured response, reduced amplitude, and prolonged latency on the left (see Figure 2). Lumbar puncture revealed increased cell count (50 cells/mmc) with normal glucose and proteins. Oligoclonal bands were found on CSF but not in serum suggesting intrathecal immune response. Cytology at that time was negative for neoplastic cells and repeated ophthalmological evaluations revealed further progression to complete visual loss in the left eye. Serum antibodies (LAC, ACA, Anti-beta2-glycoprotein ANA, anti-ds-DNA, ENA, c-ANCA, and p-ANCA) were negative. A diagnosis of possible inflammatory optic neuropathy was done. She was started on EV steroids followed by oral tapering without improvement. 

In about two months she started to complain of left hearing loss. Brainstem auditory-evoked potentials were normal on the left (see Figure 2) and audiological evaluation evidenced mild neurosensorial failure more evident on the right. She underwent contrast-enhanced cerebral MRI in October 2011 showing increased enhancement of the left optic nerve sheath. Repeated ophthalmological evaluation revealed bilateral (left > right) papilledema and she was hospitalized. Neurological examination on admission showed total blindness in the left eye with absent pupillary response to direct light stimulation. VEP revealed absent response on the left and destructured response with normal amplitude and latency on the right (see Figure 2). Cerebral and spine MRI were unchanged except for nonspecific cerebellar enhancement. She underwent repeated lumbar puncture showing further increase of white cell count (105 cells/mmc) and reduced glucose. Citology was positive for atypical epithelial cells (see Figure 1). That was diagnostic for meningeal carcinomatosis. She was referred to the Oncology Department for further evaluation and treatment.

## 3. Search Strategies and Methods

References for this review were identified through a search of PubMed from 1966 to March 8, 2012 with the terms “meningeal carcinomatosis,” “meningeal carcinomatosis and review,” “meningeal carcinomatosis and optic neuritis,” “meningeal carcinomatosis and ocular manifestations,” and “neoplastic meningitis.” Reference lists of relevant articles were also reviewed. Only articles with ocular manifestation as presenting or associated clinical features were included in our review. 

## 4. Results

We found a total of 34 papers including 33 case reports (34 patients) and 1 case series (7 patients). Information about demographic details along with clinical, instrumental, and radiological findings were recorded and summarized in Table 1. In details we looked at latency between symptoms onset and diagnosis of meningeal carcinomatosis: the mean time was 4 months, while the mean time between primary tumor diagnosis and carcinomatosis diagnosis was 20 months. Six patients (14%) had a postmortem diagnosis. 

Ocular symptoms were the presenting clinical feature in 34 out of 41 patients (83%) and were the only manifestation of meningeal carcinomatosis in 3 patients (7%). Visual loss was the presenting clinical manifestation in 17 patients (50%) and was the most common ocular symptom (70%). Visual loss was bilateral in 8 patients and unilateral in 2 patients. Sequential optic nerve involvement was observed in 5 patients. Three patients had sudden onset of blindness. Visual loss was progressive in other six patients. 

Additional ocular manifestations were diplopia (18 patients, presenting manifestation in 16 patients), ptosis (8 patients), papilledema (4), anysocoria (3), exophthalmos (2), orbital pain (2), scotomas (2), hemianopsia (1), and nystagmus (1). 

Other common symptoms were headache (24 patients), altered consciousness (10 patients), meningism (8 patients), hemiparesis (7), ataxia (7), dizziness (6), and seizures (4). V (4), VII (6), VIII (4), IX and X (2), and XII (1) cranial nerves involvement has also been reported.

Twenty-three patients underwent enhanced MRI which was diagnostic in 8 patients (35%). In 7 patients generalized meningeal enhancement was noted, and in 2 patients meningeal enhancement was associated with optic nerves enhancement, which was bilateral in 1 patient and unilateral in the other one. Three patients had cranial nerves enhancement, while isolated optic nerve enhancement was seen in only 1 patient (unilateral on the first MRI and bilateral at the second MRI). Other MRI findings included hydrocephalus (1), infarction of basal nuclei (1), and cerebellar enhancement (3). 

8 patients had normal MRI findings. 

All patients except five underwent CSF examination which was normal in 1 patient, and increased cells count was found in 11 patients (range: 9–598/mm^3^), increased protein levels were observed in 16 patients (range: 62–334 mg/dL), and decreased glucose levels were found in 8 patients (range: 5–43 mg/dL). Citology was positive in 29 patients (76%). The histology of the original tumor was highly variable with solid tumors being more frequently associated with meningeal carcinomatosis. The most common was gastric cancer followed by lymphoma, breast, and lung cancer.

16 patients underwent chemotherapy (see Table 1), and the mean life expectancy was 123 days (range 5–730 days). Autopsy was obtained in 13 patients. See Table 1.

## 5. Discussion

Meninigeal carcinomatosis occurs in 1–5% of solid tumors being adenocarcinoma the most common histology. The highest leptomeningeal diffusion rate has been reported for small cell lung cancer (11%) and melanoma (20%). Meningeal involvement occurs in about 5% of breast cancer patients [1]. Neoplastic meningitis is more likely to occur in patients with disseminated cancer, but in, respectively, 20% and 10% of cases it may manifest after a disease-free period or as a first manifestation of systemic tumors [2]. Current diagnostic methods are limited and may fail to identify MC early enough to prevent the escalation of neurological damage. Thus meningeal carcinomatosis should be included in the differential diagnosis in the presence of multifocal disease but also considered in the evaluation of isolated syndromes such as intracranial hypertension, cauda equine syndrome, or cranial neuropathy. The most common manifestation of cranial nerves involvement is diplopia due to VI cranial nerve palsy, followed by III and IV involvement. V, VIII, and optic nerve may also be affected [38].

 Diagnostic workup includes CSF examination and neuroradiological studies. The presence of malignant cells in the CSF is diagnostic, other supportive features are increased opening pressure, pleocytosis, elevated proteins, and decreased glucose levels. CSF cytology may be negative in 65% of patients on initial examination, but only in 20% of patients on second lumbar puncture [2]. CSF increased levels of vascular endothelial growth factors have been proposed as a promising biomarker with a reported sensitivity of 51–100% and a specificity of 73–100% for leptomeningeal metastases [39].

Gadolinium-enhanced MRI is also useful for the diagnosis of meningeal carcinomatosis because enhancement on MRI will reveal any irritation of leptomeninges resulting in cranial nerves or intradural extramedullary enhancement on spinal MRI.

Ocular involvement represents a frequent manifestation of meningeal carcinomatosis. The reported frequency of ocular signs may be as high as 90% [40]. The present report is an updated and systematic review of ocular manifestations of neoplastic meningitis. Our data show that ocular symptoms often represent the first clinical manifestation of meningeal carcinomatosis (83%) and the only clinical manifestation in a small proportion of subjects (7%). For this reason it should be considered in the differential diagnosis even in the absence of associated clinical symptoms more suggestive of meningeal carcinomatosis such as headache (58%), altered consciousness (24%), meningism (19%), focal signs (34%), dizziness (15%), seizures (10%), and cranial nerves other than ocular involvement (32%). The most common ocular manifestation was visual loss (70%) followed by diplopia (41%), ptosis (19%), papilledema (10%), anysocoria (7%), exophthalmos (5%), orbital pain (5%), scotomas (5%), hemianopsia (2%) and nystagmus (2%). Gadolinium-enhanced MRI was diagnostic only in about one third of patients. Meningeal enhancement was detected in about one third of patients; in two of them (25%) it was associated with focal optic nerve enhancement. In one patient (12%) contrast-enhanced MRI revealed isolated optic nerve enhancement. CSF examination was abnormal in all (36) but one patient. The most common finding was increased proteins level (44%), increased cells count (30%), and decreased glucose (22%). Citology was positive in a high proportion of patients (76%) with solid tumors being the more frequent. The first CSF examination may be inconclusive, thus if clinical and radiological suspicion persist and cell count is increased, a repeated lumbar puncture is recommended. 

## 6. Conclusion

Ocular involvement is a frequent and early clinical manifestation of meningeal carcinomatosis. Moreover clinicians should be aware that ocular involvement may mimic different diseases as shown in our case report, where neoplastic optic nerve involvement was indistinguishable from optic neuritis. Thus meningeal carcinomatosis should be included in the differential diagnosis of diplopia and visual loss in selected patients because diagnosis is often challenging.

## Figures and Tables

**Table 1:** Clinical and instrumental findings in patients with meningeal carcinomatosis (review of literature).

Study reference	Age and gender	Latency symptoms onset diagnosis	Ocular manifestations	Associated clinical features	Imaging	CSF examination	Original tumor	Treatment	Life expectancy
[4]	75, M	Not specified	Diplopia, left ptosis, anisocoria	Headache, confusion, hearing loss, VII cn (cranial nerve) palsy	CT scan (normal), MRI (GME)	(1) Prot ↑ (2) not performed	Bladder and prostate cancer	Declined	15 days

[5]	49, F	10 months	Blurred vision and diplopia, horizontal nystagmus	Dizziness, ataxia, seizures, dysarthria, VII cn palsy, left lower limb weakness	CT scan (normal), MRI + gad (FME, cauda equine, cerebellum)	(1) MTC positive (2) not performed	Ovarian cancer	WBRT. IT: topotecan (0.4 mg × 4)	4 months

[6]	39, F	6 months	Sudden bilateral visual loss, horizontal gaze palsy	headache, vertigo, seizures	MRI + gad (GME)	(1) Prot ↑, glu ↓, MTC + (2) MTC negative	Gastric adenocarcinoma	RT. IT: MTX (12.5 mg × 2/we)	—

[7]	40, F	2 months	Visual loss, bilateral sixth cn palsy, bilat papilledema	Headache, neck pain, meningism	Contrast CT scan (GMEt); MRI + gad (GME)	(1) Prot ↑, glu ↓ (2) negative	Melanoma	Not done	1 year (after symptoms onset)

[8]	33, F	11 months	VI cn palsy	Confusion, seizures, increased intracranial pressure	CT scan (FME, lateral ventricles)	Not performed	Uterine cervical neuroendocrine tumor	RT	19 months (after cancer diagnosis)

[9]	58, M	2 months (symptoms), 12 months (carcinomatosis)	Left homonymous hemianopia	Headache, ataxia	CT scan and MRI (right infarction of the caudate, internal capsule, and lentiform nucleus)	(1) MTC positive (2) not performed	Transitional cell carcinoma of the bladder	Not done	—

[10]	54, F	Postmortem diagnosis of the original tumor, 4 months (symptoms-carcinomatosis)	Ptosis, right III cn palsy	V and XII cn palsy, dysgeusia	MRI + gad (GME)	Not performed	Collecting duct carcinoma	IV: mannitol, dexamethasone, and morphinehydrochloride	3 months

[11]	65, M	2 years (carcinomatosis), 6 weeks (symptoms)	Right visual loss	Hearing loss, ataxia	MRI + gad (normal), repeated MRI + gad (FME. bilateral optic nerves, left V cn), bilateral cerebellopontine angle mass	(1) Prot ↑, glu ↓, MCT negative (2) not performed	Colorectal cancer	RT	—

[12]	61, M	Postmortem diagnosis	III cranial nerve palsy	Flaccid paraparesis with bladder retention, dysarthria, VII cn palsy, right arm weakness	MRI + gad (normal)	(1) Normal (2) Prot ↑, glu ↓, cells ↑	Lung adenocarcinoma	Not done	—

[13]	51, F	15 years (carcinomatosis), 2 months (symptoms)	Diplopia (VI cn palsy)	Paraparesis with bladder retention, peripheral neuropathy	MRI (normal)	(1) Prot ↑, MTC positive (2) MTC negative	Breast Cancer	IT: MTX (15 mg), liposomal Ara-C	Last followup: 3 years and 7 months after carcinomatosis diagnosis (clinically stable)

[14]	67, M	2 months (symptoms)	Left ptosis, diplopia, and visual loss	Headache, hemifacial sensory loss, paraplegia	MRI (GME, left retrorbital mass)	(1) Cells ↑, MTC negative (2) MTC positive	Adrenal extranodal NK/T-cell lymphoma	IV: dexamethasone	2 months (after symptoms onset)

[15]	53, M	4 months (carcinomatosis)	Intermittent diplopia	Headache, vertigo	CT scan and MRI + gad (normal)	(1) Normal (2) MTC positive	Gastric cancer	RT(45 Gy), betamethasone (16 mg/die)	3 months

[16]	43, F	156 months (carcinomatosis)	Progressive blurred vision and pain in the right eye	—	MRI + gad (FME, right optic nerve )	(1) MCT positive (2) not performed	Cervical cancer	RT + triethylenethiophosphoramide	2 months

[17]	56, M	4 days (symptoms)	III cranial nerve palsy	Headache, nausea, and low back pain	MRI + gad (normal)	(1) MTC positive (2) not performed	Lung cancer	—	—

[18]	31 (average); 3M, 4F	Average: 8 months (carcinomatosis)	Ptosis (2), diplopia (3), visual loss (3)	Headache (4), VII cn palsy (1)	MRI + gad (2 FME, cn, 2 GME, 3 normal)	(1) MTC positive (2) normal	NHL (3), ALL (1), AML (1), plasmablastic myeloma (1), breast cancer (1)	Ns	Average: 55 days

[19]	50, F	2 years (carcinomatosis)	Bilateral loss of vision	Confusion, headache	MRI + gad (thickening intraorbital optic nerves)	(1) Prot ↑, MCT positive (2) not performed	Ovarian serous cystadenocarcinoma	Ns	Ns

[20]	44, M	3 months (symptoms carcinomatosis)	Diplopia	Headache, confusion, meningeal signs, facial diplegia, ataxia, deafness	MRI (normal)	(1) Cells ↑, prot ↑, MTC positive (2) MTC negative	Lung cancer	IT: MTX (10 mg), Ara-C (39 mg). RT	182 days (after carcinomatosis diagnosis), 272 days (after symptoms onset)

[21]	67, F	6 months (carcinomatosis)	Blurred vision, diplopia	Headaches, meningeal signs, and SIADH	CT scan (normal)	(1) MTC positive (2) not performed	Gastric cancer	RT	7 months (after cancer diagnosis), 2 weeks (after carcinomatosis diagnosis)

[22]	41, M	2 weeks (symptoms)	Visual loss, left ptosis	Headache, nausea	Contrast CT scan (chiasmal thickening), MRI (hydrocephalus, increased CSF signal in the basal cisterns)	(1) Prot ↑, MTC negative (2) prot ↑, MTC negative	Rectal carcinoma	Oral: dexamethasone (4 mg)	5 days (after carcinomatosis diagnosis)

[23]	60, F	2 weeks (symptoms)	Diplopia, VI cn palsy	Ataxia, VII cn palsy, and hearing loss	CT scan (normal)	(1) Glu ↓, prot ↑, cells ↑, MTC positive (2) MTC negative	Gallbladder carcinoma	IT: MTX	2 months and 2 weeks (after symptoms onset)

[24]	—	5 months (carcinomatosis)	Bilat visual loss papilledema, exotropia, ocular pain	Hemiplegia, SAH	CT scan (normal), MRI (normal)	(1) MTC positive (2) not performed	Non-Hodgkin lymphoma	IT: MTX, corticosteroids	2 weeks (after carcinomatosis diagnosis)

[25]	49, M	Postmortem diagnosis (2 months after symptoms onset)	Visual loss, bilateral blindness	Headache, confusion, leg weakness, dysphagia, seizures, ataxia, and intermittent paralysis	CT scan (normal)	(1) Normal (2) not performed	Oesophageal adenocarcinoma	Not done	60 days (after symptoms onset)

[26]	44, M	—	Diplopia	Headache, ataxia, meningeal signs, V, VII, IX, and X cn palsy	—	(1) Cells ↑, MTC positive (2) not performed	—	IT: Ara-C.RT	—

[27]	35, F	20 weeks (carcinomatosis), 1 week (symptoms)	Bilat visual loss to blindness	Headache, vomiting, meningeal signs, and loss of consciousness	CT scan (normal)	(1) Prot ↑, glu ↓, cells ↑, MTC positive (2) prot ↑, MTC positive	Breast cancer	IT: MTX (70 mg tot), Ara-C (80 mg tot)	21 days (after symptoms onset)

[28]	36, F	NS	Blindness	Headache, loss of consciousness	CT scan (normal)	(1) MTC negative (2) MTC positive	Lung cancer	IT: MTX, Ara-C, ACNU, IL-2	2 years (after carcinomatosis diagnosis)

[29]	60, F	3 months (symptoms carcinomatosis)	Visual loss	Headaches, paraparesis	CT scan (normal), repeated CT scan of brain and orbits (right globe soft mass)	(1) Prot ↑, cells ↑ (2) prot ↑, cells ↑, MTC positive	Gastric cancer	Prednisone (80 mg)	—

[30]	37, F	—	Diplopia	Headache, nausea, vomiting, and meningeal signs	—	(1) MTC positive (2) MTC negative	Gastric cancer	IT: MTX, Ara-C, prednisolone IV: adriamycin, ftorafur	365 days (after symptoms onset)

[31]	58, M	Postmortem diagnosis	Visual loss	Vertigo, loss of consciousness	—	(1) Cells ↑ (2) not performed	Colon cancer	Not done	10 days (after symptoms onset)

[32]	11, M	—	Visual loss	—	—	—	Burkitt's lymphoma	—	—

[33]	49, M	1 month (symptoms-carcinomatosis)	Scotomas, diplopia	Headache, nausea and vomiting, and vertigo	CT scan (normal)	(1) Prot ↑, cell ↑ (2) MTC positive	Gastric cancer	IT: MTX 20 mg	60 days (after symptoms onset)

[34]	59, F	39 months (carcinomatosis)	Bilateral blindness	Dizziness, tinnitus, and confusion	CT scan (normal)	(1) Prot ↑, MTC positive (2) Pprot ↑, MTC negative. (3) prot ↑, MTC negative. (4) glu ↓, prot ↑, MTC positive.	Breast cancer	RT. IT: Ara-C (70 mg), dexamethasone. IV: 5-FU, vincristine, MTX. Oral: cyclophosphamide, prednisone	4 years and 2 months (after cancer diagnosis), 9 months (after carcinomatosis diagnosis)
	49, F	5 years and 3 months (carcinomatosis)	Bilateral blindness	—	CT scan (normal)	(1) Glu ↓, prot ↑, MTC positive (2) normal	Breast cancer	RT. IT: Ara-C, corticosteroids	5 years and 6 months (after cancer diagnosis), 3 months (after carcinomatosis diagnosis)

[35]	53, F	Postmortem diagnosis (1 month after symptoms onset)	Anisocoria, papilledema	Headache, hemiparesis, meningeal signs, and loss of consciousness	Subdural hematoma	Not performed	Gastric cancer	—	26 days (after symptoms onset)

[36]	53, M	Postmortem diagnosis (3 months after symptoms onset)	Blindness	Nausea, vomiting, confusion, ataxia, meningeal signs, and seizures	CT scan (normal)	(1) Cells ↑, prot ↑, glu ↓ (2) prot ↑	Lung cancer	Not done	3 months (after symptoms onset)

[37]	59, F	46 months (symptoms carcinomatosis); 10 months (carcinomatosis)	Scotomas, visual loss to blindness	Cerebellar ataxia	Skull radiography (normal)	(1) cells ↑, MTC positive (2) normal	Anaplastic endometrioid sarcoma	RT. IT: MTX (20 mg × 4), IM: citrovorum factor	60 days (after carcinomatosis diagnosis), 365 days (after cancer diagnosis)

Cn: Cranial nerve; SIADH: syndrome of inappropriate antidiuretic hormone secretion; SAH: subarachnoid Haemorrhage; GME: generalised meningeal enhancement; FME: focal enhancement; NHL: non Hodgkin lymphoma; ALL: acute lymphoblastic leukemia; AML: acute myelocytic leukemia; IT: intrathecal; O: oral; IM: intramuscular.
